# Prevalence and factors associated with dyslipidemia in Bangladeshi adults

**DOI:** 10.1371/journal.pone.0280672

**Published:** 2023-01-20

**Authors:** Nurshad Ali, Mitu Samadder, Rahanuma Raihanu Kathak, Farjana Islam

**Affiliations:** 1 Department of Biochemistry and Molecular Biology, Shahjalal University of Science and Technology, Sylhet, Bangladesh; 2 Department of Food Engineering and Tea Technology, Shahjalal University of Science and Technology, Sylhet, Bangladesh; Bolu Abant İzzet Baysal University: Bolu Abant Izzet Baysal Universitesi, TURKEY

## Abstract

**Background:**

Dyslipidemia is one of the modifiable risk factors for cardiovascular disease and a leading cause of morbidity and mortality worldwide. This study was designed to estimate the prevalence and factors associated with dyslipidemia in Bangladeshi adults.

**Methods:**

A total of 603 participants aged ≥ 18 years were recruited in the study. Serum levels of total cholesterol (TC), triglycerides (TG), low-density lipoprotein (LDL) and high-density lipoprotein (HDL) were analyzed using enzymatic colorimetric methods. Dyslipidemia was defined based on serum lipids levels following the standard guidelines by National Cholesterol Education Program Adult Treatment Panel III. Multivariate logistic regression analysis was applied to evaluate risk factors associated with dyslipidemia.

**Results:**

The overall prevalence of dyslipidemia was 89% with no significant difference between male (90.1%) and female (85.7) subjects. The prevalence of hypertriglyceridemia was 51.7%, hypercholesterolemia 41.6%, high LDL 43.9% and low HDL 78.8%. When participants were classified into healthy control, hypertensive and diabetic groups, the lipid levels and prevalence of lipid abnormalities were higher in hypertensive and diabetic groups compared to the control group. Low HDL level was the main prevalent dyslipidemia among study subjects. The prevalence of isolated hypertriglyceridemia, isolated hypercholesterolemia, and isolated low HDL-C was 24.7%, 14.7%, and 25.5%, respectively. Mixed hyperlipidemia was prevalent in 26.9% of the participants. According to the logistic regression analysis, significant associated factors of dyslipidemia were increased age, overweight, general and abdominal obesity, hypertension, diabetes and inadequate physical activity.

**Conclusions:**

This study shows a high prevalence of dyslipidemia in Bangladeshi adults. Important risk factors of dyslipidemia are increased age, overweight, general and abdominal obesity, diabetes, hypertension and low physical activity. Our results suggest that awareness-raising programs are required to prevent and control dyslipidemia among Bangladeshi adults.

## Introduction

Dyslipidemia is the abnormal lipid status in the blood that is defined as an elevated level of triglycerides (TG), or elevated total cholesterol (TC), or elevated low-density lipoprotein cholesterol (LDL-C), or low levels of high-density lipoprotein cholesterol (HDL-C) or a combination of these features. The global burden of dyslipidemia has increased over the past three decades and is considered an important public health issue both in low-middle-income and high-income countries [1]. Dyslipidemia is a well-known risk factor for cardiovascular disease (CVD) and related mortality in both developed and developing nations [2]. According to the World Health Organization (WHO), the global prevalence of elevated plasma levels of TC in adults aged ≥25 years was ∼39% in 2008 and more than one-third of mortality caused by CVD was attributable to raised plasma LDL-C levels [1].

In recent years, rapid urbanization, socioeconomic development, increased life expectancy, imbalanced diet, and lifestyle changes have led to a higher rate of CVD in the Southeast Asian population [3]. Bangladesh is one of the developing countries in Southeast Asia and has experienced an upward trend in the prevalence of non-communicable diseases and associated mortality over the last few decades [4, 5]. A recent literature review indicated an increasing trend in the prevalence of CVD in Bangladeshi adults higher in the urban population than rural population [6]. Several studies have reported an increased prevalence of dyslipidemia a modifiable risk factor of CVD in South Asian Countries including Bangladesh [7–11]

Screening and identification of dyslipidemia and associated risk factors are important steps to prevent the progression and incidence of cardiovascular diseases in every nation; however, there is a lack of correct statistics on the status of dyslipidemia and its determinants in the majority of developing countries [12, 13]. Data on dyslipidemia prevalence in the general population in Bangladesh is insufficient. Although, some early studies determined the prevalence of dyslipidemia in Bangladeshi adults; most of the studies were conducted at small scales and either on hypertensive or diabetic populations. Moreover, the associated risk factors of dyslipidemia in Bangladeshi populations are not well defined yet. In addition, there is a lack of data on dyslipidemia prevalence in recent years in Bangladesh. Knowing the current prevalence of dyslipidemia is an important step for enhancing awareness and for appropriate planning of health programs for preventing dyslipidemia and its adverse health effects. Furthermore, a lack of sufficient knowledge of the current status of dyslipidemia affects the implementation of appropriate programming and treatment approaches. Considering these aspects, we conducted this cross-sectional study that included healthy control, diabetic and hypertensive individuals to estimate the prevalence and factors associated with dyslipidemia.

## Methods

### Study area and study cohort

This study was conducted from January 2019 to March 2020 at the Department of Biochemistry and Molecular Biology, Shahjalal University of Science and Technology (SUST), Sylhet, Bangladesh. A total of 603 participants (general adults, academic staff members and students) were randomly recruited from the Sylhet region in Bangladesh. Diabetic subjects were enrolled at Sylhet Diabetic Hospital where they went for their regular health check-up. The diabetic participants were already diagnosed by the physicians. Inclusion criteria: (i) both sexes (ii) willingness to participate; and (ii) age ≥ 18 years. Exclusion criteria: (i) chronic inflammation; (ii) free from liver and kidney dysfunction, and infectious diseases; (iii) pregnant women and lactating mothers and (ii) participants did not complete the questionnaire and tests. The survey was approved by the Ethical Review Committee at Biochemistry and Molecular Biology Department, SUST (approval code: 02/BMB/2019). Written informed consent was obtained from the participants prior to study commencement. All methods of the study were carried out in accordance with relevant guidelines and regulations.

### Data collection

The study subject’s interviews and anthropometric and demographic data collection were performed by trained personnel. According to the standard procedures, the anthropometric information such as height, weight, waist and hip circumference (WC and HC, respectively) were measured and body mass index (BMI) was calculated [14–18]. Blood pressure was measured using a standardized automatic sphygmomanometer (Omron M10, Omron Corporation, Tokyo, Japan). The study subjects were rested for at least 10 min and then three consecutive measurements of blood pressure (BP) were taken 5 min apart. The avoid errors, the first reading of BP was discarded and the mean of 2^nd^ and 3^rd^ readings was considered for systolic and diastolic blood pressures (SBP and DBP, respectively).

### Blood sampling and laboratory measurements

Venous blood samples were obtained from the participants in the morning after overnight fasting. After centrifugation, the serum was isolated and stored at -80°C until lipid markers analysis. The levels of serum glucose, total cholesterol (TC), triglycerides (TG), low-density lipoprotein (LDL) and high-density lipoprotein (HDL) were analyzed using enzymatic colorimetric methods [19–22]. A semi-automated bioanalyzer was used to measure all the biochemical markers (Humalyzer 3000, USA). All biochemical measurements were done in one laboratory using similar methods, throughout the assay period.

### Diagnostic criteria

The lipid levels were classified following the Adult Treatment Panel III (ATP III) of the National Cholesterol Education Program guidelines [23]. Dyslipidemia was defined as having at least one of the following: TC ≥ 200 mg/dL; TG: ≥ 150 mg/dL; LDL-C ≥ 130 mg/dL and HDL-C < 40 mg/dL. Isolated hypercholesterolemia was defined as a TC ≥ 200 mg/dL and TG < 150 mg/dL; isolated hypertriglyceridemia was defined as serum TG ≥ 150 mg/dL and TC < 200 mg/dL; isolated low HDL-C was defined as HDL-C ≤ 40 mg/dL without hypertriglyceridemia or hypercholesterolemia and mixed hyperlipidemia were defined as TG ≥ 150 mg/dL and TC ≥ 200 mg/ dL. Hypertension was defined as SBP ≥ 140 mm Hg and/or DBP ≥ 90 mm Hg and/or the use of antihypertensive drugs [24–26]. Diabetes was defined following American Diabetes Association 2020 guidelines as a fasting blood plasma glucose level ≥126 mg/dL (7 mmol/L), non-fasting plasma glucose ≥200 mg/dL (11.1 mmol/L), or use of anti-diabetic medications [27, 28]. BMI was categorized into normal weight (18.5–23.0 kg/m^2^), overweight (23.1–27.5 kg/m^2^), and obesity (≥ 27.5 kg/m^2^) [29–31]. Smoking status was classified into nonsmoker and current smoker. Physical activity was defined according to the Global Physical Activity Questionnaire (GPAQ) developed by the World Health Organization (WHO) [32]. Physical activity was classified as low or sedentary, moderate, and adequate or vigorous [30].

### Statistical analyses

All the statistical analyses were performed with SPSS version 25.0 (IBM, Chicago, IL, USA). Continuous variables were presented as mean ± SD while categorical variables were presented as percentages. The differences between the groups were determined using an independent sample t-test for continuous variables and a chi-square test for categorical variables. Multivariate logistic regression analysis was used to determine the association between blood lipids and associated risk factors. In the regression model, elevated serum levels of TG, TC, HDL-C, and LDL-C were dependent variables and anthropometrics, demographics, and lifestyles were the independent variables. P values less than 0.05 are considered statistically significant.

## Results

### Characteristics of the study subjects

Characteristics of the study subjects, as stratified by gender, are presented in **Table 1**. A total of 603 participants (451 males and 152 females) aged ≥ 18 years were included in this study. The mean age of the participants was 37.9 ± 13.1 years with no significant difference between genders. The mean value for BMI showed no significant difference between genders. Males had a significantly higher mean level of TG (193.3 ± 110.6 mg/dL) compared to females (142.1 ± 88.2 mg/dL) (p<0.001). On the other hand, females had a significantly higher mean level of TC, LDL and HDL (215.3 ± 86.7 mg/dL, 151.0 ± 83.7 mg/dL and 36.6 ± 10.1 mg/dL, respectively) compared to males (198.5 ± 70.3 mg/dL, 129.0 ± 60.8 mg/dL and 32.6 ± 12.7 mg/dL, respectively) participants (p<0.05 at least for all cases). The participants were categorized into healthy control, diabetic and hypertensive based on BP and fasting blood glucose levels. 51.4% of the participants were hypertensive and 19.6% of the participants were diabetic. About 78% of the participants were used to moderate or adequate physical activities and 16% of the participants were used to smoking.

**Table 1:** Baseline characteristics of the study participants.

Variables	Total	Male	Female	P-value
N	603	451	152	-
Age (years)	37.9 ± 13.1	38.3 ± 12.9	36.6 ± 13.7	0.224
Weight (kg)	65.0 ± 11.3	67.2 ± 10.7	57.5 ± 9.9	0.000
Height (cm)	162.9 ± 9.8	165.8 ± 6.8	153.5 ± 11.8	0.000
BMI (kg/m^2^)	24.5 ± 4.0	24.4 ± 3.4	24.7 ± 5.6	0.622
WC (cm)	85.4 ± 11.2	86.2 ± 11.3	83.3 ± 10.8	0.041
HC (cm)	93.1 ± 8.4	93.1 ± 8.4	93.2 ± 8.4	0.945
SBP (mmHg)	127.3 ± 46.7	126.2 ± 14.1	130.7 ± 93.2	0.606
DBP (mmHg)	81.9 ± 10.5	82.6 ± 10.1	79.7 ± 11.6	0.011
PP (beats/min)	78.1 ± 12.5	76.5 ± 12.2	83.0 ± 12.1	0.000
Glucose (mg/dL)	117 ± 57.6	113.4 ± 55.8	124.2 ± 57.6	0.103
TG (mg/dL)	181.3 ± 107.9	193.3 ± 110.6	142.1 ± 88.2	0.000
TC (mg/dL)	202.5 ± 74.7	198.5 ± 70.3	215.3 ± 86.7	0.035
LDL (mg/dL)	134.2 ± 67.4	129.0 ± 60.8	151.0 ± 83.7	0.010
HDL (mg/dL)	33.5 ± 12.3	32.6 ± 12.7	36.6 ± 10.1	0.002
Hypertensive, n (%)	310 (51.4)	221 (49.0)	89 (58.4)	0.312
Diabetic, n (%)	118 (19.6)	74 (16.3)	44 (29.2)	0.001
Physical activity (%)				
Low	21.7	20.8	24.4	0.482
Moderate/Adequate	78.3	79.2	75.6	
Smoking (%)				0.000
No	84.1	79.2	100.0	
Yes	15.9	20.8	0.0	
Education (%)				
Illiterate	11.8	12.5	10	0.483
Primary	14.7	12.9	20	
Secondary	17.1	16.8	18	
Higher Secondary	8.9	9.3	8	
Graduate and above	47.4	48.6	44	

Data are presented as mean ± SD or %. P-values are obtained from independent sample t-test for continuous variables and Chi-square test for categorical variables. TG: Triglyceride; TC: Total cholesterol; LDL: Low-density lipoprotein; HDL: High-density lipoprotein.

### Prevalence of dyslipidemia among study subjects

**Fig 1:**
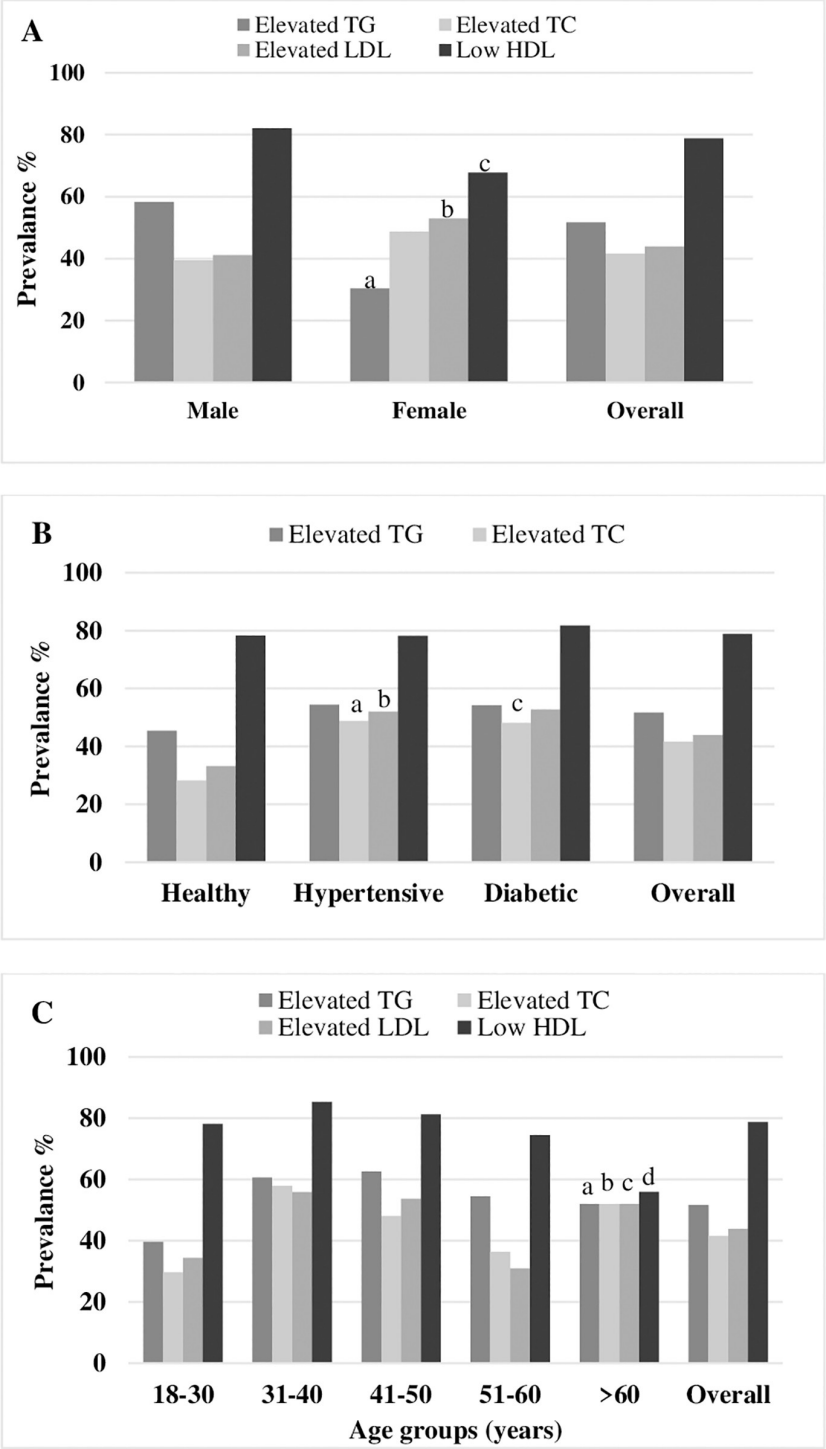
Prevalence of dyslipidemia in the gender (A), health status (B) and age (C) groups. In (A), ^a^p<0.001; ^b^p<0.05; ^c^p<0.01 when the prevalence in the female group is compared to the male group. In (B), ^a^p<0.01; ^b^p< 0.001 and ^c^p<0.05 when the prevalence in hypertensive and diabetic groups are compared to the control group. In (C), ^a,b,c^p<0.001; ^d^p<0.05 when the prevalence in the >60 years age group is compared to the lowest age group. P-values are derived from the Chi-square test.

The overall prevalence of dyslipidemia was 89% with no significant difference between male (90.1%) and female (85.7) subjects (**Table 2**). When participants were classified into healthy, hypertensive and diabetic groups, the prevalence of dyslipidemia was 84.3%, 91.8% and 88.7%, respectively (**Table 2**). The levels of lipid profile and prevalence of lipid abnormalities were higher in hypertensive and diabetic individuals than in the control individuals. Overall, hypertriglyceridemia prevalence was 51.7%, hypercholesterolemia 41.6%, high LDL 43.9% and low HDL 78.8% among the participants (**Table 3**). Low HDL levels were the main prevalent dyslipidemia among the study subjects. The prevalence of isolated hypertriglyceridemia, isolated hypercholesterolemia, and isolated low HDL-C were 24.7%, 14.7%, and 25.5%, respectively (**Table 4**). Mixed hyperlipidemia was found in 26.9% of the subjects. Hypertriglyceridemia and low HDL prevalence were higher in males, whereas, hypercholesterolemia and high LDL prevalence were higher in females (p<0.05 at least for all cases) (**Fig 1**). An increased prevalence of abnormal lipid profiles was observed in 31–40 years and 41–50 years age groups.

**Table 2:** Prevalence of dyslipidemia in different groups.

	N	Gender		Dyslipidemia (%)			
		Male	Female	Total	Male	Female	P-value
Healthy	175	137	38	84.3	86.1	75.6	0.769
Hypertensive	310	221	89	91.8	92.2	90.8	0.648
Diabetic	118	74	44	88.7	91.1	82.7	0.023
Total	603	451	152	89.0	90.1	85.7	0.133

P-values are derived from the Chi-square test. Dyslipidemia: having one or more measurements of TC ≥ 200 mg/dL; TG: ≥ 150 mg/dL; LDL-C ≥ 130 mg/dL and HDL < 40 mg/dL [23].

**Table 3:** Levels and prevalence of elevated lipid markers in different groups.

Variables	Overall	Healthy	Hypertensive	Diabetic	^a^P-value	^b^P-value
TG (mg/dL)	181.3 ± 107.9	164.2 ± 86.9	191.6 ± 120.6	179.2 ± 97.6	0.018	0.227
Elevated TG, %	51.7	45.4	54.4	54.2	0.088	0.185
TC (mg/dL)	202.5 ± 74.7	180.6 ± 48.1	217.8 ± 86.7	194.8 ± 63.8	0.000	0.052
Elevated TC, %	41.6	28.2	48.8	48.1	0.001	0.020
LDL (mg/dL)	134.2 ± 67.4	115.1 ± 46.0	147.3 ± 77.0	128.0 ± 59.5	0.000	0.061
Elevated LDL, %	43.9	33.1	52.0	52.7	0.000	0.389
HDL (mg/dL)	33.6 ± 12.3	32.3 ± 11.0	34.3 ± 13.7	33.6 ± 9.9	0.115	0.338
Low HDL, %	78.8	78.3	78.2	81.7	0.704	0.290

Data are presented as mean ± SD or n (%).^a^P value is the difference between the healthy and hypertensive group and ^b^P-value is the difference between the healthy and diabetic group. P-values for mean concentrations are derived from independent sample t-test and P-values for prevalence (%) are obtained from the Chi-square test. Elevated TG: TG ≥ 150 mg/dL; Elevated TC: TC ≥ 200 mg/dL; Elevated LDL: LDL ≥ 130 mg/dL and Low HDL: HDL < 40 mg/dL [23].

**Table 4:** Prevalence of dyslipidemia according to isolated and mixed phenotypes.

Phenotypes	Total (%)	Male (%)	Female (%)	P-value
Isolated dyslipidemia				
Isolated hypertriglyceridemia	24.7	28.8	11.3	0.000
Isolated hypercholesterolemia	14.7	10.1	29.6	0.000
Isolated low HDL	25.5	24.8	27.8	0.515
Mixed dyslipidemia	26.9	29.3	19.1	0.031

P-values are obtained from the Chi-square test. Isolated hypertriglyceridemia: TG ≥ 150 mg/dL and TC < 200 mg/dL; isolated hypercholesterolemia: TC ≥ 200 mg/dL and TG < 150 mg/dL; isolated low HDL-C: HDL-C ≤ 40 mg/dL without hypertriglyceridemia or hypercholesterolemia and Mixed hyperlipidemia: TG ≥ 150 mg/dL and TC ≥ 200 mg/dL [23].

### Multivariate logistic regression analysis for assessing the risk factors of dyslipidemia

Multivariate logistic regression models were applied to identify factors associated with lipid profile abnormalities (**Table 5**). Hypertriglyceridemia was positively and independently associated with 31–40 years, 41–50 years, and >60 years age groups, male gender, overweight, general obesity and lower education level. Hypercholesterolemia was positively associated with 31–40 years, 41–50 years, and 51–60 years age groups, overweight, general and abdominal obesity, hypertension, diabetes and low physical activity. High LDL was positively associated with 41–50 years and >60 years age groups, overweight, general and abdominal obesity, hypertension, and low physical activity. Low HDL was positively associated with all age groups, overweight, general and abdominal obesity.

**Table 5:** Multivariable logistic regression for assessing factors independently associated with dyslipidemia.

Variables	Elevated TG	P-value	Elevated TC	P-value	Elevated LDL	P-value	Low HDL	P-value
	OR (95% CI)		OR (95% CI)		OR (95% CI)		OR (95% CI)	
Age (years)								
≤30	Reference		Reference		Reference		Reference	
31–40	1.92 (1.08–3.45)	0.027	3.14 (1.69–5.83)	0.000	1.05 (0.62–1.78)	0.864	1.58 (0.92–2.71)	0.095
41–50	1.57 (0.91–2.72)	0.047	2.08 (1.13–3.80)	0.018	0.59 (0.34–1.01)	0.050	2.01 (1.20–3.36)	0.008
51–60	1.15 (0.55–2.42)	0.713	2.18 (1.02–4.66)	0.044	0.72 (0.36–1.43)	0.345	2.17 (1.07–4.38)	0.031
>60	2.75 (1.13–6.66)	0.025	1.34 (0.42–4.26)	0.618	0.10 (0.01–0.73)	0.023	6.66 (1.52–9.14)	0.012
Gender								
Female	Reference		Reference		Reference		Reference	
Male	3.28 (1.14–7.36)	0.000	0.66 (0.30–1.49)	0.319	0.49 (0.22–1.08)	0.079	2.73 (0.98–5.56)	0.054
WC (cm)								
Normal	Reference		Reference		Reference		Reference	
Increased	0.63 (0.36–1.12)	0.121	0.30 (0.14–0.64)	0.002	0.37 (0.19–0.73)	0.004	2.70 (1.00–4.76)	0.049
BMI (kg/m^2^)								
Normal	Reference		Reference		Reference		Reference	
Overweight	2.31 (0.97–4.51)	0.058	2.39 (0.88–4.05)	0.000	2.83 (1.07–4.54)	0.000	2.37 (1.64–3.15)	0.003
Obese	3.62 (2.16–5.22)	0.001	3.38 (2.57–5.79)	0.000	3.85 (1.73–5.94)	0.000	3.59 (3.87–4.27)	0.001
Hypertension								
No	Reference		Reference		Reference		Reference	
Yes	1.87 (0.63–5.57)	0.257	2.11 (1.65–3.61)	0.008	2.59 (1.73–3.38)	0.030	2.67 (0.79–9.04)	0.112
Diabetes								
No	Reference		Reference		Reference		Reference	
Yes	1.33 (0.72–2.44)	0.351	2.20 (1.24–3.93)	0.007	1.37 (0.77–2.44)	0.280	0.98 (0.50–1.94))	0.958
Smoking								
No	Reference		Reference		Reference		Reference	
Yes	1.46 (0.63–3.40)	0.376	1.80 (0.732–4.54)	0.211	1.61 (0.67–3.84)	0.283	2.08 (0.69–6.29)	0.194
Physical activity								
Moderate/Adequate	Reference		Reference		Reference		Reference	
Low	1.32 (0.70–2.53)	0.397	1.24 (0.78–1.85)	0.049	1.25 (0.80–1.94)	0.045	1.09 (0.49–2.44)	0.836
Education								
Illiterate	Reference		Reference		Reference		Reference	
Primary	0.38 (0.06–1.68)	0.033	0.98 (0.41–2.34)	0.972	0.82 (0.37–1.83)	0.630	1.65 (0.61–4.43)	0.318
Secondary	0.80 (0.36–1.76)	0.578	1.11 (0.49–2.58)	0.792	1.37 (0.63–2.95)	0.424	0.84 (0.35–2.02)	0.704
Higher Secondary	0.69 (0.27–1.80)	0.453	1.03 (0.39–2.73)	0.960	1.08 (0.44–2.65)	0.866	2.43 (0.70–8.43)	0.163
Graduate and above	0.90 (0.46–1.74)	0.749	1.65 (0.81–3.36)	0.168	1.45 (0.75–2.81)	0.272	1.18 (0.55–2.55)	0.668

Values are presented as OR (95% CI). OR = Odds ratio, CI = Confidence Interval. Multivariate logistic regression was applied to evaluate the relationship between elevated lipid profile markers and associated factors.

## Discussion

The findings of this study show that dyslipidemia is widely prevalent in Bangladeshi adults. We estimated the prevalence of dyslipidemia and associated risk factors in healthy control, hypertensive and diabetic populations. The overall prevalence of dyslipidemia was 89% of which 84.3%, 91.8% and 88.7% in the control, hypertensive and diabetic groups, respectively. Overall, the prevalence of hypertriglyceridemia was 51.7%, hypercholesterolemia at 41.6%, high LDL at 43.9% and low at HDL 78.8% among the participants. Among the study subjects, the most prevalent dyslipidemia was low levels of HDL-C. Both lipid markers level and the prevalence of lipid markers abnormalities were higher in diabetic and hypertensive subjects compared to the healthy control subjects.

The prevalence of CVD is a growing epidemic throughout the world with dyslipidemia being one of its major modifiable risk factors. Some previous studies indicated that the increased prevalence of CVD among the South Asian population is primarily due to the pattern of dyslipidemia [33]. Population surveillance is an important step in identifying and monitoring the risk factors for CVD; however, in Bangladesh, there is insufficient community-based data on the prevalence of dyslipidemia and CVD. A previous survey conducted in 2005–2011 in a sub-urban population of Mymensingh district in Bangladesh reported the prevalence of dyslipidemia as 17.8% of high TG, 16.9% of high TC, 15.7% of high LDL-C and 8.8% of low HDL [8]. In our study, the overall prevalence of hypertriglyceridemia was 51.7%, hypercholesterolemia 41.6%, high LDL 43.9% and low HDL 78.8% which is high than those reported in the previous study [8]. Another previous study by Chowdhury et al estimated the levels of lipid profile among subjects (aged 30–60 years) who went to the National Centre for Control of Rheumatic Fever and Heart Disease (NCCRF&HD) in Dhaka, for routine health check-ups [3]. The authors reported an elevated level of lipid profile markers in hypertensive individuals than in normotensive individuals, although the prevalence of dyslipidemia was not presented in the study. Similar to this study findings [3] we also found a higher level of lipid profile among hypertensive subjects than in the normotensive subjects. One of the major limitations of that study [3] was that the participants were recruited from an urban hospital which actually does not represent all hypertensive patients in Bangladesh. A further study conducted in the Noakhali District, a southern part of Bangladesh, reported the prevalence of dyslipidemia among diabetic patients recruited from two diabetic hospitals [34]. The results showed the prevalence of dyslipidemia at 73% in males and 71% in females, with high TG at 41.96%, high TC at 35.42%, high LDL at 71.33% and low HDL at 49.70%. In our diabetic participants, the overall prevalence of dyslipidemia (88.7%) and individual lipid abnormalities (high TG 54.2%, high TC 48.1%, high LDL 52.7% and low HDL 81.7%) were higher than those reported by Das and Bank except for high LDL [34]. Another study conducted in the Kushtia region in Bangladesh reported comparatively a lower prevalence of dyslipidemia in newly diagnosed type 2 diabetic patients 72.6% in males and 75.7% in females [35]. However, the prevalence of individual lipid abnormalities in that study (high TG 57.6%, high TC 52.3%, high LDL 50% and low HDL 74.2%) [35] was close to the prevalence level observed in our study.

Some studies in our neighbouring countries like India and Pakistan also reported the prevalence and factors associated with dyslipidemia in the general population. For example, in a study by Joshi et al, the overall prevalence of dyslipidemia was 79%, hypertriglyceridemia 29.5%, hypercholesterolemia 13.9%, high LDL-C 11.8% and low HDL-C 72.3% in Indian population [10]. A study in Pakistan reported a prevalence of dyslipidemia of 98.1% in diabetic subjects, 97.3% in pre-diabetes subjects and 95.2% in the non-diabetic population which is slightly higher than those found in our study [36]. In that study 48.9% of participants had hypertriglyceridemia, 39.3% had hypercholesterolemia, and 39.7% had high LDL-C levels while 83.9% of men and 90% of women had low levels of HDL-C [36]. In our study and the other studies in our neighbouring countries, the most prevalent dyslipidemia was low HDL levels, although the exact reasons are not clear why low HDL-C level is highly prevalent in the South Asian population, further studies are needed to get insights into it. It has been suggested that high levels of HDL-C play protective effects by reducing the cholesterol entry rate into the cells and increasing the cholesterol release rate from cells by promoting reverse cholesterol transport via scavenging excess levels of cholesterol from peripheral tissues [37]. Furthermore, high levels of HDL-C also inhibit LDL-C oxidation and atherogenic effects of oxidized LDL-C through its anti-inflammatory and antioxidant properties. It has been shown that an increase of 1 mg of serum HDL-C correlates with the reduction of coronary heart disease risk by 2% in men and 3% in women [38]. However, the protective roles of HDL-C in CVD in the South Asian population appear to be little compared to the other ethnic population. Whether low levels of HDL-C are a real risk factor for increased cardiovascular risk in South Asians remains unclear, and further studies are needed to get into the insights.

On the other hand, TG to HDL-C ratio is considered an inflammatory and metabolic predictor. Some studies indicated high TG to HDL-C ratio levels as a useful marker in predicting non-alcoholic hepatosteatosis and poor blood pressure control [39, 40]. It has also been suggested that the uric acid to HDL-C ratio is associated with poor blood pressure control and could be a predictor of diabetic control in men with type 2 diabetes [41, 42]. The increased burden of dyslipidemia in hypertensive and diabetic patients and related complications can be reduced by the management of hypertension and diabetes. Further research is needed to determine the management of dyslipidemia and related complications. Adequate knowledge of various factors controlling hypertension and diabetes and their complications is important. An unhealthy diet, poor management strategies, treatment and lifestyle are the possible leading causes of dyslipidemia and related complications.

In the present study, the prevalence of dyslipidemia was comparatively higher in males than in females, although some lipid markers levels (TC, HDL-C and LDL-C) were higher in female participants. A higher prevalence of dyslipidemia was also found in males in the previous study in Bangladesh [34] and Pakistan [43]. Although, there are some other studies where an opposite finding ie., a higher prevalence of dyslipidemia was found in females than in their counterparts [35, 36]. An increasing trend of lipid abnormalities with age was observed among our participants. Similar findings were found in a study conducted in India [10]. Increased prevalence of dyslipidemia in females with ages might be related to menopausal transition and loss of estrogen, which may act as a triggering factor and increase metabolic dysfunction [44, 45]. However, considering the management of lipid profile, attention should also pay to males especially in the middle age 31–50 and females >50 years in both screening and intervention of dyslipidemia in Bangladeshi adults since these subgroups are at more risk of having dyslipidemia.

The present study shows that increased age, general and abdominal obesity, hypertension, diabetes and inadequate physical activity were significant risk factors for dyslipidemia. The link between dyslipidemia and the risk of CVD is well proven and the tendency for certain CVD risk factors to be clustered, such as insulin resistance, obesity, glucose intolerance, dyslipidemia and hypertension, referred to as the metabolic syndrome [46]. It is well known that obesity is one of the major contributors to dyslipidemia. In the present study, overweight, general and central obesity were the significant risk factors for dyslipidemia. Similar findings were reported in previous studies [47–50]. Furthermore, low or inadequate physical activities were associated with the risk of dyslipidemia in our study. This was in accordance with early studies [49, 51, 52]. Some intervention studies also showed that adequate exercise as physical activity can improve lipid profile status, resulting in a reduction in TG levels and an increase in HDL-C levels [53, 54]. Thus, a proper community-based prevention strategy focusing on behavioural changes, especially promoting regular physical activities would be useful to control and reduce dyslipidemia.

Finally, our study results along with some early studies indicated that dyslipidemia prevalence is increasing at an alarming rate in the Bangladeshi population. Therefore, appropriate comprehensive national health policy should be implemented on a priority basis. The associated risk factors of dyslipidemia can be reduced by increasing awareness, implementation of proper public health policy, management of hypertension and diabetes and a healthy lifestyle. A major strength of our study was that it provided important information regarding the prevalence and associated factors of dyslipidemia both in healthy and diabetic and hypertensive individuals. However, there were some limitations in our study which should be taken into consideration. Firstly, we did not have data on the food habits of the participants, therefore we could not analyse the dietary factors associated with dyslipidemia. Secondly, our study was a cross-sectional design, thus, cannot determine causality. Thirdly, the sample size in the present study was relatively small, therefore, our findings do not represent the entire population of the country. Further investigations especially longitudinal studies are needed to get deep insights into the prevalence and risk factors of dyslipidemia among the Bangladeshi population.

## Conclusions

This study found a higher prevalence of dyslipidemia among the study participants. Low HDL was the most frequent form of dyslipidemia among the participants, followed by an elevated level of TG. Increased age, general and abdominal obesity, diabetes, hypertension and inadequate physical activity were significant risk factors for dyslipidemia. Our findings suggest the need for screening programs for assessing blood lipid levels as well as effective intervention programs targeting associated risk factors reduction and prevention of adverse effects of dyslipidemia. Routine measurement of blood lipids needs to be included in the treatment of hypertensive and diabetic individuals for optimum health care. Furthermore, lipid-lowering drugs and anti-diabetic drugs should be affordable to patients to avoid sudden complications.
